# Primary care behind the former “Iron Curtain”: changes and development of primary healthcare provision in the Eastern part of the European Union

**DOI:** 10.1017/S1463423619000410

**Published:** 2019-09-09

**Authors:** Csilla Semánová, Sarolta E. Rurik, Csaba Dózsa, Zoltán Jancsó, László R. Kolozsvári, Anna Nánási, Markéta Pfeiferová, Imre Rurik

**Affiliations:** 1 Department of Family and Occupational Medicine, University of Debrecen, Debrecen, Hungary; 2 Independent Macroeconomic adviser, Budapest, Hungary; 3 Faculty of Health Care, University of Miskolc, Miskolc, Hungary; 4 Department of General Practice, First Faculty of Medicine, Charles University, Prague, Czech Republic

**Keywords:** Alma-Ata declaration, eastern bloc, economy, family medicine, healthcare, international, iron curtain, medicine, primary care

## Abstract

**Background:**

The Alma-Ata Declaration was a big step in the development of primary care, defining the main tasks and populations’ expectation. Celebrating the 40th year’s anniversary is a good opportunity to make an analysis. Development of primary care was not parallel in the Eastern and Western part of Europe.

**Aim:**

To provide an overview on the societal and economic situation, structural and financial changes of healthcare systems in the former ‘Soviet bloc’ countries, to present an analysis of the primary healthcare (PHC) provision and to find relationships between economic development and epidemiological changes of the respective countries.

**Method:**

Epidemiological data, healthcare expenditures and structure, and financing schemes were compared; systematic literature search was performed.

**Results:**

Visible improvements in population health, in the national economic condition, structural changes in healthcare and more focus to primary care were experienced everywhere. Higher life expectancies with high inter-country variation were observed in the former ‘Soviet bloc’ countries, although it could not be clearly linked to the development of healthcare system. PHC provision improved while structural changes were rarely initiated, often only as a project or model initiation. Single-handed practices are yet predominant. The gate-keeping system is usually weak; there were no effective initiatives to improve the education of nurses and to widen their competences. Migrations of workforce to Western countries become a real threat for the Central-East European countries.

**Conclusion:**

Lack of coordination between practices and interdisciplinary cooperation were recognized as the main barriers for further improvement in the structure.

## Introduction

Primary care could be defined as the first level of medical provision, where patients present their health problems and where the majority of the population’s curative and preventive health needs could be fulfilled (Starfield, 1994; Kringos *et al.*, 2015). Primary care should be provided close to where people are living and without obstacles to access. In medical term, primary care is a generalist medical care, focusing on the person as a whole, instead of on only one specific organ system or health problem (Kringos, 2012; Schäfer, 2016).

Primary care was firstly defined as one of the health services levels in the Dawson Report from the United Kingdom in 1920. Hundred years ago the other two levels were also defined: secondary health centres and teaching hospitals (Lord Dawson of Penn, 1920). Doctors, who are active in this line of work, have been designated as primary physicians, family physicians or general practitioners (GP) depending on the country they are working in.

Europe was always ahead in primary care. The European Union of General Practitioners (UEMO) was established in 1967 by six countries [uemo]. In 1972, the World Organization of Family Doctors (WONCA) was founded by member organizations in 18 countries [WONCA]. During decades, it becomes a worldwide professional network, a prosperous scientific organization with strong political influence. Later on, other continental professional organizations and networks were also established [European General Practice Research Network (EGPRN), European Forum for Primary Care (EFPC)].

In 1978, the World Health Organization (WHO) initiated a common thinking about primary care; the scope was re-defined during the International Conference held in the former Soviet Union. The *Declaration of Alma-Ata* was soon affirmed at the World Health Assembly’s meeting in May of 1979 and had a wide international impact (Declaration of Alma-Ata, 1978; World Health Organization, 1981).

Family medicine and primary healthcare (PHC) made major advances since the *Alma-Ata Declaration* (De Maeseneer, 2017). What was initially seen as a sound principle – to structure healthcare from the community level in response to the needs of individuals and population – turned out to be a determining factor of efficient, safe and timely healthcare (Starfield, 1994). Later on, the WHO analysed the developments of the previous three decades, and in the World Health Report of 2008 WHO recognized PHC as a core-component of health systems (World Health Organization, 2008).

Up to around 1990, countries of Central and Eastern Europe were economically and politically dominated by the Soviet Union. There was a common market of strict economic and industrial cooperation (*COMECON*), as well as a close military alliance system (*The Warsaw Treaty Pact*). Healthcare in all countries was a public responsibility. Organization, management and delivery of care were provided by the state or local municipality authorities. Financing and administration were bureaucratic and strongly centralized. PHC was provided as public service by under-qualified recent graduates or by other specialists who were sent from hospitals to serve in primary care without adequate clinical specialization in family medicine. This profession had a low respect among other medical specialists (Windak and van Hasselt, 2005; Rurik and Kalabay, 2009). Nearly all inhabitants were entitled to access to the healthcare system free of charge. Healthcare was financed from general taxation by the state. Patients were allocated to local providers according to their place of residence. There was no ‘gate-keeping’ function; patients had easy or even unlimited access to most outpatient clinical specialists, often to inpatient services as well. Healthcare providers (both doctors and nurses) were underpaid; getting a low salary, therefore, informal payment (‘tipping’ or ‘parasolvence’) was widespread to obtain better access or higher-quality services. There were little or no contribution by patients to funds of healthcare services, except for medicines; the scope of services was poorly defined. A healthcare provision was not always allocated to the needs of patients or local population (Jack *et al.*, 1997; Windak and van Hasselt, 2005; Győrffy *et al.*, 2017).

In the previous century, development of PHC services differed widely by countries. Western countries recognized the importance of primary care earlier and they had made changes in their healthcare system accordingly. The former ‘Eastern bloc countries’ needed more changes in their healthcare system. The ‘change of regimes’ followed by democratic elections and establishing new governments, as political requirements, usually was not enough. Structural and financial changes were also required before implementing appropriate PHC provisions (Rurik and Kalabay, 2009).

After the collapse of the Soviet-Union, some of its former member countries (*Estonia, Latvia*, and *Lithuania*) became independent. *Slovenia* and *Croatia* were separated from the former Yugoslavia, in the early 1990s, before the bloody ‘Balkan war’ erupted. The states of Czechoslovakia also parted company in 1993; the *Czech Republic* and *Slovakia* were formed and embarked on their own historical and economical paths. *Bulgaria, Hungary, Poland* and *Romania* initiated democratic changes in the society and started reforms of the economy and the healthcare system.

## Aim

We try to focus to the Eastern part Europe, to the other side of the former ‘Iron Curtain’, where development of PHC was different in the ‘Socialist’ time.

Our paper is an attempt to provide a brief overview of the societal, economical situation of healthcare systems focusing on primary care, to present an analysis of the structural and financial changes of PHC provision and to find relationships between the structure of PHC, economic development, epidemiological changes and governmental health policies of the respective countries.

## Method

Macroeconomic data of healthcare expenditures and financing schemes of the respective countries were searched (European Commission, 2018; Organisation for Economic Cooperation and Development, 2018; World Health Organization, 2018). Data were explained among the results.

Databases of national and international organizations were accessed to compare epidemiological data and reports on the structure of healthcare system (European Commission, 2015; The World Bank, 2018a, b).

The recognition and position of primary care in the respective countries were analysed based on the systematic literature searches performed in the European Primary Care Activity Monitor (PHAMEU) study (Kringos *et al.*, 2015).

A self-structured questionnaire (*see* Appendix) was sent to the chairpersons of WONCA-affiliated national primary care associations/societies and national representatives within the EGPRN network, at least for two persons in each country (European General Practice Research Network, 2018; World Organization of Family Doctors, 2018). The most important and relevant answers were presented and discussed.

## Results

In all the upcoming tables the abbreviations of respective countries were used as in their internet domain, like: BG (Bulgaria), CR (Croatia), CZ (Czech Republic), Estonia (EE), HU (Hungary), LV (Latvia), LT (Lithuania), PO (Poland), RO (Romania), SI (Slovenia) and SK (Slovakia).

### Healthcare expenditures

EUROSTAT, the OECD and the WHO have long been cooperating to create a common data collection on health expenditures. The main results of this cooperation are the *International Classification for Health Accounts* (ICHA), a *Joint Questionnaire on Health Expenditure* and the manuals ‘*A System of Health Accounts* (SHA)’ (European Commission, 2018; Organisation for Economic Cooperation and Development, 2018; World Health Organization, 2018).

Health expenditure can be divided by functions of healthcare, excluding capital investment. They define the *Total Current Healthcare Expenditure* (CHE) as the sum of all healthcare, expenditure on activities of services on curative, rehabilitative and long-term care, ancillary services, medical goods, preventive care, governance and health system and financing administration, and unknown other healthcare services. Health expenditure can also be divided by *financing schemes* of healthcare: *government* and/or *compulsory contributory* healthcare financing schemes, *voluntary* healthcare payment schemes, *household out-of-pocket* payment and unknown financing schemes.

In Table 1, the governmental and compulsory healthcare expenses were presented with the voluntary and household/out of pocket payments. Because of differences between national regulations and reporting systems, these could be variable.

**Table 1.** Total CHEs as percent of national GDP and their distribution between financing schemes

	BG	CR	CZ	EE	HU	LV	LT	PL	RO	SK	SI
***1990***											
**GDP (PPP) USD**	5191	*	12 666	*	6321	*	*	6179	5262	*	*
**Total healthcare spending – % of GDP**	N	*	3.7	*	5,2	*	*	4.3	*	*	*
**Gov+comp.** **Vol+H outofp**	N	*	3.6	*	*	*	*	3.9	*	*	*
	N	*	|0.1|	*	*	*	*	|0.4|	*	*	*
***2000***											
**GDP (PPP) USD**	6377	10 753	16 188	942	11 876	8018	8456	10 651	5877	11 355	18 048
**Total healthcare spending – % of GDP**	5.9	7.7	5.7	5.2	6.8	7.9	5.8	5.3	4.2	5.3	5.7
**Gov+comp.** **Vol+H outofp**	3.5	6.6	5.2	4.0	4.7	4.0	4.0	3.7	3.4	4.7	5.7
	|2.4|	|1.1|	|0.6|	|1.2|	|2.1|	|3.9|	|2.0|	|1.7|	|0.8|	|0.6|	|2.1|
***2010***											
**GDP (PPP) USD**	14 949	19 240	27 694	21 603	21 556	17 576	20 110	21 069	17 027	24 987	27 766
**Total healthcare spending – % of GDP**	7.1	8.1	6.9	6.3	7.6	8.6	6.8	6.4	5.7	7.8	8.6
**Gov+comp.** **Vol+H outofp**	3.9	6.9	5.8	4.8	5.1	5.2	4.9	4.6	4.6	5.6	6.3
	|3.2|	|1.2|	|1.2|	|1.5|	|2.5|	|3.4|	|1.9|	|1.8|	|1.1|	|2.2|	|2.3|
***2017***											
**GDP (PPP) USD**	20 329	25 264	36 916	31 638	28 375	27 598	32 093	29 291	25 841	32 110	34 802
**Total healthcare spending – % of GDP**	n	n	7.1	6.7	7.2	6.3	6.3	6.7	n	7.1	8.3
**Gov+comp.** **Vol+H outofp**	n	n	5.8	5.1	4.8	3.4	4.2	4.6	n	5.7	6.0
	n	n	|1.3|	|1.6|	|2.5|	|2.9|	|2.2|	|2.0|	n	|1.4|	|2.3|

(Government + compulsory and household/out of pocket)

**Gov+comp. =** Government + compulsory healthcare **|**

**Vol+H outofp =** Voluntary schemes healthcare spending + household out of the pocket

**n =** not yet available

* Data are lacking where no reliable or discrepant sources were available and from countries that were yet in the phase of becoming independent.

*In the OECD data bank Bulgaria, Croatia and Romania are missing, as they are not members*.

### Mortality-based indicators

The life expectancies improved significantly in all countries. In the previous 25 years, the highest increase was registered among the Slovenian males (nine years), the lowest (three years) among the men in Bulgaria and Lithuania. Differences between these countries become wider.

Among women, the lowest improvement was registered also in Bulgaria (three years), the highest (seven years) also in Slovenia, the same as females living in the Czech Republic and in Estonia. Data of these countries are presented in Table 2.

**Table 2.** Changes in life expectancies at birth, among male and female

Life expectancy at birth [years]	BG	CR	CZ	EE	HU	LV	LT	PL	RO	SK	SI
***1990***											
**Male**	68	69	68	65	65	64	66	67	67	67	69
**Female**	75	76	75	75	74	75	76	76	73	75	77
***1995***											
**Male**	67	68	70	61	65	60	63	68	66	68	70
**Female**	75	77	77	74	75	73	75	76	73	76	78
***2000***											
**Male**	68	69	72	65	67	65	67	70	68	69	72
**Female**	75	77	78	73	76	76	78	78	75	77	79
***2005***											
**Male**	69	72	73	67	69	66	65	71	68	70	74
**Female**	76	79	79	78	77	77	77	79	76	78	81
***2010***											
**Male**	70	74	74	71	71	69	68	72	70	72	76
**Female**	77	80	81	81	78	78	79	81	77	79	83
***2015***											
**Male**	71	74	76	73	72	70	69	74	72	73	78
**Female**	78	80	82	82	79	80	80	82	79	80	84

### Structure and workforce of healthcare system

Number of hospital beds decreased significantly, more visible in the ‘Baltic’ states as presented in Table 3. There were only small changes in the number of medical doctors in these countries. Among nurses, a slight increase was observed in three countries (Czech Republic, Hungary and Slovenia) and a decrease in Slovakia.

**Table 3.** Numbers of hospital beds, active medical professionals (doctors and nurses) per 1000 inhabitants

	BG	CR	CZ	EE	HU	LV	LT	PL	RO	SK	SI
***Numbers*** **Total, per 1000 inhabitants**											
***1990***											
**Hospital beds**	9.8	7.4	9.9	11.6	10,1	13.4	12.5	n	8.9	n	6.0
**Doctors**	3.2	2.1	2.7	3.5	2.9	3.5	n	2.1	1.8	n	n
**Nurses**	n	n	7.3	8.1	5.2	5.7	n	5.5	n	n	n
***1995***											
**Hospital beds**	10.4	5.8	8.5	8.3	9,2	11.2	10.9	n	7.6	n	5.7
**Doctors**	3.5	2.0	3.0	3.2	3.0	2.8	3.7	2.3	1.8	n	n
**Nurses**	n	n	7.4	6.4	5.3	4.9	8.9	5.5	n	n	n
***2000***											
**Hospital beds**	7.4	6.2	7.8	7.0	8.2	8.8	8.8	n	7.4	7.9	5.4
**Doctors**	3.4	2.4	3.4	3.1	2.7	2.9	3.6	2.2	1.9	3.4	2.1
**Nurses**	n	n	7.6	5.8	5.3	4.6	7.6	5.0	n	7.4	6.8
***2005***											
**Hospital beds**	6.4	5.5	7.6	5.4	7.9	7.9	7.3	6.5	6.6	6.8	4.8
**Doctors**	n	2.5	3.6	3.1	2.8	3.0	3.7	2.1	n	3.0	2.4
**Nurses**	n	n	8.1	6.3	6.0	5.0	7.3	5.1	n	6.0	7.5
***2010***											
**Hospital beds**	6.5	5.6	7.0	5.3	7.2	5.7	7.2	6.6	6.3	6.5	4.6
**Doctors**	3.8	2.9	3.6	3.2	2.9	3.1	4.0	2.2	2.5	3.4	2.4
**Nurses**	n	n	8.1	6.1	6.2	5.0	7.4	5.3	n	6.1	8.2
***2015***											
**Hospital beds**	n	n	6.5	5.0	7.0	5.7	7.0	6.6	n	5.8	4.5
**Doctors**	n	n	n	3.4	3.1	3.2	4.3	2.3	n	3.5	2.8
**Nurses**	n	n	8.0	6.0	6.5	4.7	7.7	5.2	n	5.7	8.8

n: Data are lacking where no reliable or discrepant sources were available

#### Strength and position of primary care

The dimensions of primary care were broken down into a number of key attributes; country data on all indicators were transformed into scores indicating the level of primary care orientation of healthcare systems, ranging from 1 (*low* primary care orientation) to 3 (*high* primary care orientation) and are presented in Table 4. There were differences between scores as seen in rows. The ratio of expenses for public health and prevention within the whole healthcare budget was the highest in Romania and Slovakia (it is given in percent) (Kringos *et al.*, 2015).

**Table 4.** Strength and position of primary care scored by dimensions

Dimensions	Scores by countries										
	BG	CZ	CR*	EE	HU	LV	LA	PL	RO	SK	SI
Total governance of primary care	2.46	2.37		2.5	2.2	2.44	2.48	2.32	2.52	2.3	2.51
Total economic conditions of primary care	1.88	2.03		2.06	2.1	2.08	2.08	2.06	2.18	2.1	2.2
Total primary care workforce development	1.95	1.9		2.2	2.1	1.8	2.14	1.92	2.07	1.8	2.23
Total access to primary care scores by country	2.15	2.4		2.2	2.4	2.3	2.16	2.4	2.25	2.25	2.45
Total continuity of primary care scores by country	2.35	2.44		2.45	2.4	2.3	2.42	2.4	2.3	2.4	2.3
Total coordination of primary care score by country	1.4	1.65		1.7	1.45	1.95	1.65	1.85	1.55	1.35	1.8
Total comprehensiveness of primary care score by country	2.55	2.3		2.4	2.3	2.4	2.6	2.3	2.2	1.95	2.4
Level of gate keeping	3	2		3	2.5	2.5	3	2	3	2	3
*Prevention and public health expenditures of total healthcare expenditures [%]*	*3.5*	*2.2*		*2.6*	*2.4*	*1.2*	*1.3*	*2.2*	*5.9*	*4.5*	*4.1*

* Croatia was not included in this study.

### Structure and competence of primary care

According to the questionnaires used for evaluation, approximately 18–25 % of all active doctors are working in the primary care.

Migrations of healthcare professionals were mentioned as a challenge for all countries. The main target destinations are Germany, the Scandinavian countries and the UK.

The mean age of practicing family physicians is usually high (52–58 years), except Lithuania (45 years).

The numbers of providers are usually balanced: increasing in Romania and Lithuania and decreasing in Hungary.

There are existing networks for paediatric care in almost all countries, where the even higher mean age of doctors were also mentioned as problem.

The average practice size is different, the numbers of enrolled patients are the lowest 1300 (in Lithuania), and the highest are 1800 (in Romania and Croatia).

In all countries, the GPs are mainly contracted with the national health insurance funds as private enterprisers (mainly self-entrepreneurs) or having own companies (Ltd) or are working as employees of public or private employers. One-third of GPs are working as public servants in Croatia.

Because of different contracting and remuneration systems, it is hard to define their real income; it varies between from 1100 EUR (Lithuania) to 4000 EUR, monthly (Croatia) after taxation.

Single-handed practices are still predominant; one doctor with one employed nurse.

Group practices or professional cooperation between providers is still in initiative phase in some countries (Hungary, Romania) but are dominantly working in Lithuania. Some business chains are buying more and more practices (Czech Republic). In some countries there is cooperation under the umbrella of healthcare centers (Poland, Croatia).

PHC finances are usually based on *capitation*, with some corrections with *fee for service* elements, without significant financing based on *quality indicators*.

The education of nurses usually remained within the former framework. There are no nurse practitioners in the system; nurses did not get extended competencies.

Gate-keeping functions are declarative only; secondary care could be reached directly more often.

### Postgraduate and continuous medical education

Family medicine is a recognized medical specialty in all countries; universities/medical schools have already established their departments of family medicine. Vocational training lasts four years in Croatia and Romania and three years in the others. Only half of the family physicians are professionally certified in Croatia, while the majority of GPs in other countries have already passed the national board exams.

Programmes to complete continuous medical education are organized by the universities and professional associations, rarely NGOs.

Privatization and an opportunity for independent work, overtaking some, but not enough competences, recognition as a discipline were mentioned as the main advantages of primary care reforms in almost all countries. Administrative tasks were usually mentioned as a barrier to better performance.

Lack of coordination between practices and interdisciplinary cooperation were recognized as the main barriers for further improvement.

Family physicians are often involved in preventive activities and screening motivated by extra financing.

In almost all countries, primary physicians are respected members of the society.

## Discussion

### Main findings

Visible improvements in population health, and in the national economic condition, structural changes in healthcare, more focus to primary care were experienced everywhere. Higher life expectancies with high inter-country variation were observed in the former ‘Soviet bloc’ countries, although it could not be clearly linked to the development of healthcare system. PHC provision were improved, while structural changes were rarely implemented, often only as a project or model initiation. Migrations of workforce become a real threat.

### Life expectancies

It could be supposed that improvement in living conditions due to the increase of GDP contributed to similar changes in life expectancies, without any clear statistical relation. Improvement of medical services and in the technology could also facilitate these changes, independently from the structure of primary care.

### Economic conditions

The economic positions improved differently in these countries, mainly depending on the respective national policies, traditions and cooperation. Economic conditions of primary care are largely determined by the proportion of total health expenditure spent on primary care and the financial conditions for access to care for patients. Cost sharing and co-payment can threaten equity in financial access to care (Győrffy *et al.*, 2017; Organisation for Economic Cooperation and Development, 2018). In these countries, the out of pocket payment or co-payment is usually higher than in the ‘Western’ part of Europe (Organisation for Economic Cooperation and Development, 2018; World Health Organization, 2018). Primary care professionals can be salaried or self-employed providers, either contracted or not to the health services or health insurance system. The employment status and mode of remuneration may also influence the attractiveness of primary care professions. It could explain the differences in the income of GPs within and between these countries.

No significant relationship was found between the national income (GDP) of countries and their overall economic conditions of primary care. This suggests that the financial policies and mechanisms applied are of greater influence than the financial resources available (Kringos *et al.*, 2015).

Primary care expenditure strongly varies among countries. We were unable to acquire reliable data about the ratio of primary care financing within the whole healthcare budget. To some extent, this results from the services included in the expenditures for primary care. A uniform methodology for calculating primary care expenditure across countries is not available and this hampers the comparability of this indicator. For example, in some countries it is limited to costs for family practice only, while in others freely accessible specialist care services are also included. Costs for community nursing, primary mental healthcare, dentistry and emergency care may be included in primary care costs. Even in family practice fund-holding, elements for laboratory tests and other investigations can be included (Kringos *et al.*, 2015; Schäfer, 2016).

### Organization of healthcare and inter-professional collaboration

Supported by technological innovation, hospital stays become generally shorter or avoidable by ambulance services and one-day surgeries; therefore, the number of hospital beds was decreased in all countries. More complex care could be provided in the community. Strong primary care is often associated with the gate-keeping position of GPs; however, the strength of primary care is based on more other characteristics (Kringos *et al.*, 2013). Majority of the health problems can be handled within primary care. If not, GPs are expected to guide the patient through the referral process to a medical specialist or hospital. The gate-keeping system is usually weak in these countries or often symbolic, reported by the experts who were questioned. There were no effective initiatives in the education of nurses and the widening of their competences. Other professional contributors are not yet involved in the primary care. Chronic conditions and multi-morbidity can be treated more effectively by different closely collaborating healthcare workers among whom tasks may be redistributed. An integrated primary care level has a major role to play, preferably in relation to community and occupational services. In these countries primary care consists mainly of GPs working in single-handed practices. Only initiatives for group practices were mentioned, not similar like partnership in the UK. In France, where there is a tradition of single-handed practice, a national plan has been successful in increasing the number of group practices and multidisciplinary *maisons de santé* in primary care (Afrite, 2013). There were no national plans mentioned in these countries, but only some initiatives. In Hungary, a primary care model programme ran between 2012 and 2016, supported by the Swiss Government to enlarge the focus of PHC for public health issues, focusing better to the prevention (Rurik, 2014; Sándor *et al.*, 2013). The implementation area of the *Swiss Contribution* was in an economically and socially deprived region of Hungary. The implemented screening programmes improved the level of PHC provision of this area (Nagy *et al.*, 2018; Sándor et al., 2018a, b). These data differ from previous Hungarian studies (Simay *et al.*, 2005; Jancsó *et al.*, 2006; Ilyés *et al.*, 2012; Torzsa *et al.*, 2017).

### Limitations

- We did not have an access to reliable data of PHC expenditures of the countries examined.
- The relationship between GDP, healthcare expenditures and life expectancies is not clear; it could be influenced by other societal or environmental factors as well.
- Some of the indicators used in this paper (numbers of hospital bed, doctors and nurses) are not strongly characteristic of the system of respective national healthcare provision.
- There is no measurable information how governments of the respective countries handled the PHC system.
- There is no available basement to compare how structure of primary care provision can influence the outputs of population’s health.

Primary care provision should manage the ageing of the European population, the changing health threats and morbidity, workforce developments and growing possibilities of technology as well. Further challenges include the growing prevalence of non-communicable diseases and the increase of more complex demand, resulting from higher rates of multi-morbidity. Meanwhile there are significant improvements in the technological background like eHealth, telemedicine and point of care testing that offering new service models in primary care too. Healthcare systems, that are traditionally designed to manage acute episodes of a single illness, need a more integrated provision of services in healthcare facilities as well as in the community (Kringos, 2012). There is a need to improve collaborations and professional organizations offer a good platform for it [efpc,egprn, wonca].

The position of GP in most Central and Eastern European countries is formally adequate, but a lot of efforts are still needed to achieve the desired level of its recognition and quality (Švab *et al.*, 2004). Within the former Eastern bloc countries, only Slovenia was mentioned among those countries that had relatively strong primary care system (Kringos *et al.*, 2013).

Primary care is essential to effective, sustainable healthcare systems. The success of each country’s healthcare system depends upon the adequacy of its primary care system (Gree *et al.*, 2005). This seems to be the most relevant message of the Alma Ata Declaration, even 40 years later.

## APPENDIX

**Table:**

Human resources		
Actual **number** of **GPs**		
Trends in the number of GPs?	Decreasing/increasing	
No of GPs for paediatric care		
Does it exist a different network for the care of children?		
Average size of the GP practice (No. patients/practice)		
Age cohort of GPs	mean…… year	
Number of primary care nurses	in a practice	
How are they educated?	Do they receive any other education?	
What competencies they have?	Does ‘nurse practitioners’ existing? Did it start any new type of education giving more competencies for nurses?	
**Structure, financing**		
**Status** of GPs	Private/public servant	
If private, how is the contractor?	National fund/private insurance Co-s?	
If public servant, who is the employer?		
How are GPS financed?	Based on salary/on capitation fee for services? Please describe?	
Average ***netto*** income of GPs? (after deducted with taxes) EUR/range: from - to		
GPs are working…… Please provide ratio, if available?	in single-handed practice	in group practices?
Do they employ or contract other health professionals?	if yes, please list them	
Is the ‘gate-keeping function’ of the PC strong?	give examples	
Do they perform any activities, as *prevention*? (primary, secondary)	Is this activity financed?	
According to your knowledge, which percent of national healthcare budget is spent for primary care?	….% please provide relevant website	
**Education**		
Is PC an academic discipline? Existing departments of Family Medicine at the medical faculties?	If yes, since when? after 1990? before 1990? Recent number of departments	
Is there a *vocational training* for family physicians?	Since when? How long is it? ….years	
Does it exist a *board examination* of family physicians?	Since when?How is the ration of board certified family physicians in the PC system?	
is it an *obligatory **CME*** for GPs	Organized by the universities?	
**Organization**		
Are any PC *organization*(s) in your country?	Please provide name and link to the website	
Are any *Institute* of primary care	Operated by the government? By universities?	
Please describe the main tasks of this institution		
**Additional personal remarks**		
Please write about, how is the respect of PC in the whole population?		
How strong is the governmental support for primary care?	Only verbal visible	
Describe the most important changes	After 1990/after joining the EU?	
What changes in PC system could be considered as the consequences of EU membership?		
The most important and predictable challenges for the national PC system in the future?		
Is any visible migration of GPs toward other EU member countries - migration of primary care nurses?	Which are the target countries?	
Any other relevant remarks	Give link to a relevant websites	
